# Sources of Male and Female Students’ Belonging Uncertainty in the Computer Sciences

**DOI:** 10.3389/fpsyg.2019.01740

**Published:** 2019-08-13

**Authors:** Elisabeth Höhne, Lysann Zander

**Affiliations:** Division of Empirical Educational Research, Institute of Education, Leibniz Universität Hannover, Hanover, Germany

**Keywords:** belonging uncertainty, ability-related stereotypes, social identity, minority students, higher education, STEM, computer science, gender

## Abstract

Belonging uncertainty, defined as the general concern about the quality of one’s social relationships in an academic setting, has been found to be an important determinant of academic achievement and persistence. However, to date, only little research investigated the sources of belonging uncertainty. To address this research gap, we examined three potential sources of belonging uncertainty in a sample of undergraduate computer science students in Germany (*N* = 449) and focused on (a) perceived affective and academic exclusion by fellow students, (b) domain-specific academic self-efficacy beliefs, and (c) perception of one’s individual performance potential compared to that of fellow students in the field. Perceived affective and academic exclusion by fellow students and domain-specific academic self-efficacy beliefs were significant predictors of female students’ uncertainty about belonging in computer science. The perception of one’s individual performance potential in comparison to that of fellow students, however, was a relevant predictor of both male and female students’ belonging uncertainty in computer science. Our findings imply an expanded view of the theoretical concept of belonging uncertainty that goes beyond mere concerns of social connectedness.

## Introduction

“I remember walking into one of the classes at Stanford and just deciding not to take the class because I was one of only three women there, and I just felt so intimidated.” The experience that former co-president of Women in Computer Science at Stanford University, Catherina Xu, publicly expressed in 2017 is a feeling that students from stigmatized and underrepresented social groups frequently experience in academic settings. When minority group members question their fit in an educational environment, a state of belonging uncertainty can emerge and manifest, which has been found to adversely affect academic domain identification, achievement, persistence, and career aspirations (Woodcock et al., 2012; Cundiff et al., 2013; Walton et al., 2015; Höhne and Zander, in press). Although there is a growing body of research on the consequences of belonging uncertainty, to date, only little research has empirically investigated the sources of the feeling that “people like me” do not belong (Walton and Cohen, 2007). The current research addresses this gap and examines the perceived exclusion by fellow students, domain-specific academic self-efficacy beliefs, and the perception of one’s individual performance potential in the domain compared to that of fellow students as sources of belonging uncertainty.

In the university context, one group that is likely to experience belonging uncertainty are female students in STEM (science, technology, engineering, and mathematics) domains, who, in Germany as well as in many other Western industrial nations, constitute the numerical minority in these stereotypically male-connoted domains (OECD, 2017). We chose the domain of computer science because with a percentage of 18.35%, it is currently one of the subjects with the lowest rate of female students in Germany (status winter term 2017/2018; Federal Statistical Office of Germany, 2018). Furthermore, in contrast to other STEM fields, computer science recently underwent a significant decrease in the number of female first-year students (−8.8% female students vs. −2.7% male students in winter term 2017/2018 as compared to the previous academic year; Federal Statistical Office of Germany, 2017).

Identifying and understanding the sources of belonging uncertainty is not only relevant to provide important cues for creating a stimulating and encouraging learning environment for females studying a traditionally male-dominated subject. In a broader sense, it is crucial to explore possible barriers and its antecedents to IT professions to prevent one of the fastest growing economic sectors from an intensification of skills shortage (European Institute for Gender Equality, 2017).

## Theoretical Framework

### Belonging Uncertainty and Its Consequences in Academic Environments

The need to form and maintain positive and stable interpersonal relationships, i.e., the need to belong and to feel socially connected, is a fundamental human motivation (Baumeister and Leary, 1995). In educational environments, students’ experienced sense of belonging has been empirically shown to positively affect achievement motivation, performance, well-being, and retention (Walton and Cohen, 2011; Good et al., 2012; Walton and Carr, 2012).

Negative competence-related stereotypes, such as the belief that women lack ability in quantitative fields (Smeding, 2012), can convey the message that people of certain social groups are less qualified, accepted, and valued. As a consequence, negatively stereotyped students who constitute a numerical minority in their respective academic domain might doubt their belongingness and experience a state of belonging uncertainty (Walton and Cohen, 2007, 2011; Good et al., 2012; Smith et al., 2013). Conceptually, this mental and emotional state takes the form of a hypothesis that guides individuals’ perception and interpretation of various social contexts and is not restricted to specific social groups, settings, or contexts (Walton and Cohen, 2007).

Noteworthy, belonging uncertainty differs from the phenomenon of stereotype threat, which describes the psychological predicament of being at risk to confirm a negative stereotype about one’s own social group (Steele, 1997). Stereotype threat is evoked in specific, high-stake performance situations. Here, negatively stereotyped students underperform because they anticipate others’ low expectations and negative performance-related feedback based on their group membership, ultimately confirming stereotypes they sought to avoid (for two meta-analyses see Nguyen and Ryan, 2008; Appel et al., 2015). To doubt one’s belongingness to an academic domain, individuals do not need to experience a situation of evaluation, to anticipate or actually receive negative feedback on a specific task (Mallett et al., 2011). Instead, belonging uncertainty can manifest itself in the absence of a concrete performance situation and describes a more general concern that can give rise to the feeling that “people like me do not belong here” (Walton and Cohen, 2007, p. 83).

Uncertainty about one’s belongingness in an academic environment can have a number of negative consequences. Experiences of rejection based on membership in a devalued group can lead individuals to anxiously expect future rejections, which, in turn, can lower their sense of well-being and negatively influence their relationships with peers and professors (Mendoza-Denton et al., 2002). Female students were also shown to have greater anticipatory self-doubts about their abilities and expectations of unfairness when being preoccupied with concerns and expectations about rejection based on the social category of their gender (London et al., 2012). Continuously doubting one’s belongingness in academic contexts can further contribute to actual decrements in intellectual performance (Mendoza-Denton et al., 2002) and lead stigmatized individuals to lower their identification with the scientific discipline (Spencer et al., 1999; Davies et al., 2002; Deemer et al., 2016). By disidentifying from the threatening domain, i.e., by removing the domain as a basis of self-evaluation as adaption strategy, negatively stereotyped students can uphold and maintain their feelings of self-worth (Steele, 1997; Spencer et al., 1999). Because the identification with a domain is an important predictor of career motivation (Schuster and Martiny, 2017), it can have detrimental effects on both personal and societal level when students of marginalized or stigmatized social groups disidentify with certain academic domains and, as a long-term consequence, abstain from scientific careers (Woodcock et al., 2012; Cundiff et al., 2013).

### Potential Sources of Belonging Uncertainty

Although previous findings have demonstrated that belonging uncertainty is an important determinant of academic achievement and persistence, only little research has been dedicated to the investigation of its sources.

The theoretical conceptualization of belonging uncertainty as concern about the quality of one’s social ties and connectedness (Walton and Cohen, 2007) draws on the hypothesis that human beings have a pervasive need to belong that is reflected in their desire to have positive relationships with others (Baumeister and Leary, 1995). As social beings, we rely on interdependencies with conspecifics, wherefore the perception of not being socially accepted and integrated is experienced as aversive. Within social pain theory, the aversive emotional state of social exclusion is even described as unpleasant as the experience of suffering physical pain because it signals the probability of being socially excluded or isolated, which constitutes an evolutionary disadvantage (Eisenberger et al., 2003; MacDonald and Leary, 2005). Among the negative consequences of social exclusion are cognitive impairments as well as significant mental and physical detriments, including higher rates of morbidity and mortality (Berkman and Syme, 1979; Baumeister et al., 2002; Uchino, 2006; Cohen and Janicki-Deverts, 2009).

In challenging, achievement-oriented, and competitive environments, people are suggested to be sensitive to the quality of their social bonds, and members of negatively stereotyped social groups are found to be even more susceptible to feelings of social belonging uncertainty (Walton and Cohen, 2007). The results of an experimental study by Walton and Cohen (2007) substantiate this assumption: a manipulation leading individuals to believe they would only have few friends in the domain of computer science decreased Black but not White students’ sense of belonging to that domain. Thus, only individuals afflicted with a threatened social identity, i.e., the part of the self-concept that is based upon social group membership (Tajfel and Turner, 1986), seem to be vulnerable to subtle situational cues that signal a lack of social connectedness (Murphy et al., 2007; Murphy and Taylor, 2012). We therefore expected students who perceived that they were excluded from social activities and academic exchange with fellow students to be more uncertain about their belonging within the domain of computer science – but that this relationship would be stronger for female students.

A slightly modified manipulation targeting academic ability in a quantitative domain, however, negatively affected female students’ sense of belonging. Here, students were asked to list either two skills or eight skills they had in the domain of computer science, with the result that female but not male students rated their social fit to that domain lower in the eight skills than in the two skills condition (Walton and Cohen, 2007). To the extent that the experienced difficulty of the task was presumed to increase with the number of skills that had to be generated (see also Schwarz et al., 1991; Hermann et al., 2002), the findings indicate that female students were more sensitive to information relevant to their insecurity, i.e., information about their quantitative ability, eventually leading to larger decrements in their perceived sense of belonging. Belonging uncertainty may therefore not only grow with doubts about one’s social connectedness, but also with doubts about one’s abilities and competencies in a discipline. Experimental research on induced feelings of belonging, either in a social or in an academic domain related to university, further supports the idea that there is a difference between social and academic belonging. A study by Skourletos et al. (2013) experimentally manipulated feedback on a measure students completed and found that minority students’ performance on an IQ test was significantly higher when they were told they had the academic potential and ability to do well scholastically than when they were told that they had social potential. Because the results indicated that the performance deficit in minority students caused by negative stereotypes could not be remedied by an attributed social potential and by telling students that people with similar scores were involved in various social organizations at university, Skourletos et al. (2013) suggested a difference between academic and social belonging. This also corresponds to the results of a study by Lewis and Hodges (2015), who found a negative correlational relationship between social belonging and a measure of ability uncertainty. Therefore, it seems to take both positive social interactions within a domain and a sense of relative fit regarding one’s academic competencies in order to feel one belongs.

To approach minority students’ uncertainty about their academic abilities, we focus on academic self-efficacy beliefs, which have been linked to a wide range of desirable scholastic outcomes, such as students’ achievement and college retention (Robbins et al., 2004; Valentine et al., 2004; Parker et al., 2014). Within social cognitive theory, self-efficacy beliefs refer to a person’s confidence in his or her ability to accomplish certain tasks (Bandura, 1997), and several career-related decisions are influenced by our judgments of self-efficacy (Lent et al., 1994). While people tend to pursue tasks and approach domains in which they feel capable, they avoid those which they believe exceed their abilities (Bandura, 1997). With regard to our target group, much research has shown that females tend to have lower levels of self-efficacy in quantitative fields even when they perform equally well or when they outperform their male counterparts (for a meta-analysis see Huang, 2013). Further, previous experimental studies have indicated the adverse influence of stereotype activation on self-efficacy beliefs (Hoyt and Blascovich, 2007; Lerdpornkulrat et al., 2012; Wang et al., 2017). Because female students have to contend with a negative stereotype about their quantitative ability, we argue that the discrepancy between females’ confidence in their ability and their actual performance can be attributed to ability-related stereotypes that are firmly grounded in society. It is therefore plausible that low academic self-efficacy beliefs are a source of female students’ feelings of belonging uncertainty, which we expected in the present study.

Furthermore, research has shown that students’ competence beliefs are influenced by the frames of reference that they use to evaluate themselves. According to the “Big-Fish-Little-Pond-Effect” (BFLPE; Marsh and Parker, 1984), students compare their academic ability with that of their classmates – a process that affects the development of students’ academic self-concept (Marsh et al., 2007). Thus, a student among low-achieving classmates would show a higher academic self-concept (a “big fish in a little pond”) than an equally able student among high-achieving classmates (a “little fish in a big pond”) – an effect that has been replicated in different academic domains and across a large number of culturally and economically diverse countries (Seaton et al., 2009).

Even though the concepts of academic self-efficacy and academic self-concept both describe perceptions of the self in academic contexts, they differ in that self-efficacy refers to an individual’s convictions to be able to succeed in specific academic tasks (Bandura, 1997), whereas self-concept refers to the perception of one’s general academic abilities and skills in a domain (Marsh, 1987; Trautwein et al., 2009). For example, the expectation that one can succeed in a study-related task (e.g., to pass an exam in computer science) is an efficacy judgment, however, it is not a judgment of whether one is competent in this domain in general (e.g., to be a successful computer scientist or a “computer science person”). Whereas self-concept is formed through experiences with and interpretations of one’s social environment (Shavelson et al., 1976), frame-of-reference effects are not central to self-efficacy beliefs and should be largely eliminated in students’ responses to self-efficacy measures (Marsh et al., 2019).

Although academic self-concept has empirically been found to have consistent reciprocal effects with achievement and educational attainment (Marsh and O’Mara, 2008), it does not necessarily accurately reflect actual achievement. With a view to females in STEM subjects, previous research found that female students, on average, have lower academic self-concepts in mathematics- and science-related domains – even when they perform on the same level as their male peers (Ludwig, 2010; OECD, 2015). A study by Ertl et al. (2017) could further demonstrate that stereotypes about females’ interests, abilities, and need for conformance in STEM directly affect the academic self-concept of female STEM students. Moreover, while upward comparisons in the context of attainable achievement can serve as inspiration (Lockwood and Kunda, 1997) and benefit performance (Blanton et al., 1999), social comparisons in the presence of negative stereotypes seem to work via different psychological mechanisms. Here, upward comparisons with in-group members are enhancing because the superior other’s performance is challenging the negative ability-related stereotype about one’s own social group, whereas the opposite holds in case of upward comparisons with out-group members, who constitute most of the salient targets of comparison in historically homogeneous environments (Blanton et al., 2000). Given the negative ability-related stereotype about females and their numerical underrepresentation in the male-dominated STEM domains, we argue that females’ comparisons of their academic ability with that of their – predominantly male – classmates can cause feelings of belonging uncertainty in terms of academic fit.

### The Present Research

Comprehensively understanding the factors that explain female students’ lower academic domain identification and retention in STEM-related subjects is crucial to remedying growing gender disparities in computer science. To gain a better understanding of the situation of female students in computer science and the concept of belonging uncertainty, the present study examined potential sources of male and female students’ uncertainty about belonging in the domain of computer science. In light of relevant previous findings, we expected that (a) the perceived affective and academic exclusion by fellow students, (b) domain-specific academic self-efficacy beliefs, and (c) the perceived individual performance potential in comparison to that of fellow students in computer science would predict female but not male students’ sense of belonging uncertainty in the academic domain of computer science.

## Materials and Methods

### Participants

A total of 453 computer science students participated in the study. Four students did not provide any information on our variables of interest, including their gender, and were excluded from the analyses. Our final sample consisted of 449 students (345 male, 104 female) with an average age of 22.03 years (*SD* = 4.36). Participation rate across both time points of assessment was high with an overall percentage of 94.47%.

### Procedure

The study was conducted in winter term 2016/2017 and 1 year later at the Institute of Computer Science at a large German university (more than 30.000 enrolled students), from which permission was obtained beforehand. Questionnaires were distributed in the mandatory functional programming classes of the first-year students, which have to be attended at least 80% of the time in order to pass. Moreover, students have to actively take part in the tutorial classes (max. 30 students), e.g., by handing in weekly exercises that they worked on in groups of two. Data was collected in 26 tutorial groups with an average size of 18.00 students (*SD* = 4.89). Each of the tutorial groups was assessed at two time points: at the beginning of the lecture period in week two or three (T1) and four weeks later (T2).

All students in the functional programming classes were asked to participate in a study on their “first impressions and experiences at university” and were told that the aim of the study was to “improve the conditions of studying.” Students were also informed about their voluntariness of participation, assured of their anonymity, and instructed that all of their data would be kept confidential and be used for research purposes only. In addition, students gave their written consent on top of the questionnaire at each time point of assessment and were informed of their right to withdraw their participation at any time of the study without giving any reason. All participants generated a personal code, allowing us to match the questionnaires of each one person while ensuring an anonymous data processing. Once students gave their consent, research assistants emphasized that there were no right or wrong answers and encouraged participants to answer in whatever way seemed right for them.

The completion of the paper-and-pencil questionnaire took between 15 and 20 min. At the first assessment, students participated without receiving any reward or compensation. With the objective of increasing the motivation and incentive to participate in the second assessment, students received sweets and could participate in a raffle in which they could win a book voucher. In order to take part in the raffle and to be informed about the results of the study, students could write their email address on a separate list after the assessment.

### Measures

#### Belonging Uncertainty

An adapted version of a measure by Walton and Cohen (2011) was used to assess students’ subjective level of uncertainty about their belonging within computer science. The original scale of Walton and Cohen (2011) consisted of three items, however, a factor analysis found that one of the items loaded weakly on the common factor. The remaining 2-item scale demonstrated a good internal consistency (α = 0.820), wherefore we decided to apply it in our study. Because students’ degree of uncertainty about their social belonging within the particular domain of computer science was of interest in our study, rather than their belonging to the entire college, the content of the two-item measure was adapted to the subject of study: (1) “Sometimes I feel that I belong to this study program, and sometimes I feel that I don’t belong to this study program^1^ “ and (2) “When things don’t go well, I often think that maybe I don’t belong to this study program.” Students indicated their agreement on a 5-point Likert scale (1* = strongly disagree*, 5 *= strongly agree*), with greater values reflecting a higher level of belonging uncertainty. Both items were found to be internally consistent (α = 0.712), summed, and averaged into a composite score. The intraclass correlation (ICC) for students within tutorial groups was determined to be 0.001.

#### Affective and Academic Social Exclusion

Students’ perceived exclusion from non-academic social activities with fellow students (affective exclusion) and subject-related exchange with fellow students (academic exclusion) was assessed using a self-developed scale consisting of four items (e.g., “Sometimes I have the feeling that other students meet privately and I am not included,” “I have already noticed that other students engage in subject-related exchange and I am not included”; for the full scale see section “Supplementary Material”). The reason why we used a self-developed measure was that there was no adequate measure for our targeted population in terms of age and the university context which encompassed both students’ perceived social and academic exclusion. All items used a 5-point Likert response scale (1 = *strongly disagree*, 5 = *strongly agree*) and formed a reliable scale (α = 0.890).^2^

#### Domain-Specific Academic Self-Efficacy

Students’ confidence in their ability to succeed in study-related tasks, even when confronted with difficulties or under challenging circumstances, was assessed using an adapted version of a German scale by Jerusalem and Schwarzer (1986). Whereas the original scale consists of seven items altogether, a shortened two-item version was applied here: (1) “I am confident that I have the competencies to perform well in this subject” and (2) “I can cope with difficult situations and challenges in my studies when I try hard.” The corresponding items used a 5-point Likert response scale (1* = strongly disagree*, 5* = strongly agree*), for which reliability analysis revealed a sufficient internal consistency (α = 0.700).

#### Performance Potential Compared to Fellow Students

To tap into the social comparison impression that has been shown to influence students’ academic self-concept, an adapted measure by Walton and Cohen (2007) was used to assess students’ perception of their individual performance potential compared to the potential of their fellow students. In contrast to the original measure, students were prompted to think about their fellow students before being asked to evaluate their individual potential to succeed in their studies compared to their peers on a percentile scale: “I have more potential than … of the students in this subject” (10% = *more potential than 10% of the students*, 90% = *more potential than 90% of the students*, in steps of 10%).

#### Academic Performance

Since our sample primarily consisted of first-year university students (71.9%) who had not received any grades at university yet, we assessed a proxy variable of previous academic performance of the respondents by asking them to report their average grade obtained in the German school-leaving examination.

#### Socio-Demographic Data

Participants were asked to provide information on their gender and age. To prevent priming effects based on gender, which could impact students’ reports of their sense of belonging uncertainty (Mallett et al., 2011), socio-demographic data were assessed at the very end of the questionnaire.

### Statistical Analyses

Data analyses, if not stated differently, were run using Mplus version 8.1 (Muthén and Muthén, 1998–2017). At first, descriptive statistics and bivariate correlations for all variables of interest were calculated. In addition, mean differences and standardized mean differences^3^ between male and female students were computed. Prior to running our main analyses, we conducted Little’s MCAR test (Little, 1988) within SPSS’s Missing Value Analysis option (version 25.0; IBM Corp, 2017) to examine missing data patterns in our sample. This test is implemented as a chi-squared test with the null hypothesis that cases of missing data are missing completely at random (Little and Rubin, 1989). We then estimated missing values in Mplus using full information maximum likelihood estimation (FIML), which has been proven to be superior to other missing-data techniques, such as list- or pairwise deletion, mean substitution, or last observation carried forward, with respect to model estimation, bias, and efficacy (Schafer and Graham, 2002; Peugh and Enders, 2004). In order to avoid listwise deletion of individuals with missing data on *x*-variables, independent variables were treated as dependent variables within Mplus as a result of specifying the means and variances of the independent variables (Hox et al., 2015). Because students (level-1 unit) were nested in tutorial groups (level-2 unit), which may violate the assumption of independent observations within regression analyses (e.g., Nimon, 2012; Snijders and Bosker, 2012), we used the TYPE = COMPLEX command in Mplus to take into account the hierarchical data structure and to adjust the standard errors. The multiple linear regression was conducted using a robust maximum likelihood (MLR) estimator. In the main model, we regressed belonging uncertainty at T2 on the T1 variables perceived affective and academic exclusion, domain-specific academic self-efficacy, perceived relational performance potential as well as each predictor’s interaction with gender, while controlling for belonging uncertainty at T1 and students’ previous academic performance. Significant interactions were visualized using the web-based data visualization tool interActive (McCabe et al., 2018). Because our analyses focused on effects within persons and because we expected the relevant reference group for participants to be students in their tutorial group, rather than all students in their study program^4^, all level-1 variables, except the categorical variable of gender, were entered group-mean centered into the model. Accordingly, slopes are interpreted as the increase in the criterion variable associated with one unit increase in the predictor variable – relative to the tutorial group’s mean (for the implication of different centering choices in terms of interpretation see Park, 2008).

## Results

The descriptive statistics for our dependent and independent variables are shown in Table 1. Means and standard deviations are presented for the total sample as well as separately for male and female students. In addition, gender differences on all relevant variables were examined using linear regression analyses with a dummy variable taking a value of zero for male students and one for female students. Significant mean differences were found for belonging uncertainty at both time points of assessment, domain-specific academic self-efficacy, the perceived performance potential in relation to fellow students, and previous academic achievement. Female students, on average, reported higher levels of belonging uncertainty in computer science than male students both at T1 (*B* = 0.515, *p* ≤ 0.001, *d* = 0.445) and T2 (*B* = 0.443, *p* ≤ 0.01, *d* = 0.402). Regarding the perceived affective and academic exclusion by fellow students, no significant mean difference between male and female students was found (*B* = −0.022, *p* = 0.840, *d* = 0.020). By contrast, male students reported higher levels of academic self-efficacy in computer science (*B* = −0.245, *p* ≤ 0.01, *d* = 0.341), and evaluated themselves, unlike female students, as above average regarding their own performance-related potential in comparison to that of other students in the field (*B* = −0.949, *p* ≤ 0.001, *d = 0.*480). Interestingly, female students, however, reported higher school-leaving examination grades and thus, a better academic performance in high school than male students (*B* = −0.318, *p* ≤ 0.05, *d* = 0.189).

**TABLE 1:** Means, standard deviations, and mean comparisons by gender of the dependent and independent variables.

		**Belonging uncertainty**	**Social exclusion**	**Academic self-efficacy**	**Relative potential**	**Belonging uncertainty**	**Academic performance**
		**T2**	**T1**	**T1**	**T1**	**T1**	**T1**
							
	***N***	***M* (*SD*)**	***M* (*SD*)**	***M* (*SD*)**	***M* (*SD*)**	***M* (*SD*)**	***M* (*SD*)**
Total	449	2.76 (1.11)	2.88 (1.08)	3.95 (0.73)	5.04 (2.01)	2.82 (1.16)	2.37 (1.67)
Males	345	2.66 (1.07)	2.89 (1.09)	4.00 (0.71)	5.25 (1.79)	2.71 (1.16)	2.44 (1.90)
Females	104	3.07 (1.16)	2.86 (1.05)	3.75 (0.74)	4.24 (2.00)	3.20 (1.15)	2.12 (0.63)

*B* (*SE*)		0.443 (0.17)	−0.022 (0.11)	−0.245 (0.10)	−0.949 (0.21)	0.515 (0.15)	−0.318 (0.14)
Sig.		0.010^∗∗^	0.840	0.015^*^	0.000^∗∗∗^	0.001^∗∗∗^	0.019^*^

All values were estimated using Mplus and full information maximum likelihood estimation (FIML). Standard errors were adjusted for the hierarchical data structure. Mean values (*M*), standard deviations (*SD*), and standard errors (*SE*) were rounded to two decimal places. Regression coefficients (*B*) were rounded to three decimal places. Gender: 0 = male, 1 = female. ^*^p ≤ 0.05; ^∗∗^*p* ≤ 0.01; ^∗∗∗^*p* ≤ 0.001.

In Table 2, bivariate correlations are shown. All predictor variables, except students’ previous academic performance, which we controlled for in the subsequent regression analyses, correlated significantly with our criterion. While social exclusion and gender (males = 0, females = 1) positively correlated with belonging uncertainty, negative correlations were obtained for domain-specific academic self-efficacy and perceived potential in relation to fellow students. Since there were weak to moderate correlations between some of the explanatory variables, multicollinearity was tested by means of variance inflation factors (VIFs), applying a cut-off value of 10 (Bühner and Ziegler, 2009). VIFs were examined within SPSS (version 25.0; IBM Corp, 2017), based on a multiple regression analysis of belonging uncertainty on all independent variables. With the lowest VIF-score being 1.026 and the highest being 1.714, no significant inflation of standard errors due to non-orthogonality among the predictors was indicated.

**TABLE 2:** Correlations of the dependent and independent variables.

	**1**	**2**	**3**	**4**	**5**	**6**	**7**	**VIF**
1 Belonging uncertainty T2	1	0.176^∗∗^	–0.475^∗∗^	–0.428^∗∗^	0.681^∗∗^	0.182	0.167^*^	–
2 Social exclusion		1	−0.124^*^	–0.052	0.207^∗∗^	0.037	–0.008	1.051
3 Academic self-efficacy			1	0.545^∗∗^	–0.467^∗∗^	–0.037	−0.142^*^	1.714
4 Relative potential				1	–0.401^∗∗^	–0.083^∗^	–0.199^∗∗^	1.521
5 Belonging uncertainty T1					1	0.062^∗^	0.185^∗∗^	1.537
6 Academic performance						1	–0.079	1.026
7 Gender							1	1.086

*N* = 449. Values were estimated using Mplus and full information maximum likelihood estimation (FIML). Standard errors were adjusted for the hierarchical data structure. VIF = variance inflation factor of the independent variables (variables 2–7; results were estimated using SPSS). Values were rounded to three decimal places. Gender: 0 = male, 1 = female. ^*^p ≤ 0.05; ^∗∗^*p* ≤ 0.01; ^∗∗∗^*p* ≤ 0.001.

According to Little’s MCAR test, which showed that missing data in our sample ranged from 2% to 38% with an overall proportion of 14%, these data points were missing completely at random (χ^2^ = 76.72, *df* = 68, *p* = 0.219), indicating that the probability of missingness does not depend on any observed or missing values.

To test the core assumption of our research that perceived exclusion by fellow students, domain-specific academic self-efficacy, and perceived performance potential compared to others are relevant predictors of belonging uncertainty within computer science, while controlling for students’ previous academic achievement and the initial level of belonging uncertainty, a multiple linear regression analysis was conducted (see Table 3).

**TABLE 3:** Multiple linear regression for variables at T1 predicting belonging uncertainty at T2.

**Explanatory variables**	***B***	***SE***	***β***	***p***
Social exclusion	–0.015	0.045	–0.014	0.744
Academic self-efficacy	–0.099	0.082	–0.063	0.233
Relative potential	–0.084	0.030	–0.147	0.005^∗∗^
Belonging uncertainty	0.540	0.036	0.561	0.000^∗∗∗^
Academic performance	–0.050	0.123	–0.074	0.724
Gender	0.029	0.129	0.011	0.823
Social exclusion X gender	0.204	0.096	0.087	0.038^*^
Academic self-efficacy X gender	–0.424	0.175	–0.133	0.019^*^
Relative potential X gender	0.019	0.058	0.017	0.742

N = 449. All values were estimated using Mplus and full information maximum likelihood estimation (FIML). Standard errors were adjusted for the hierarchical data structure. Values were rounded to three decimal places. SE = standard error of B. Gender: 0 = male, 1 = female. ^*^p ≤ 0.05; ^∗∗^*p* ≤ 0.01; ^∗∗∗^*p* ≤ 0.001.

**FIGURE 1:**
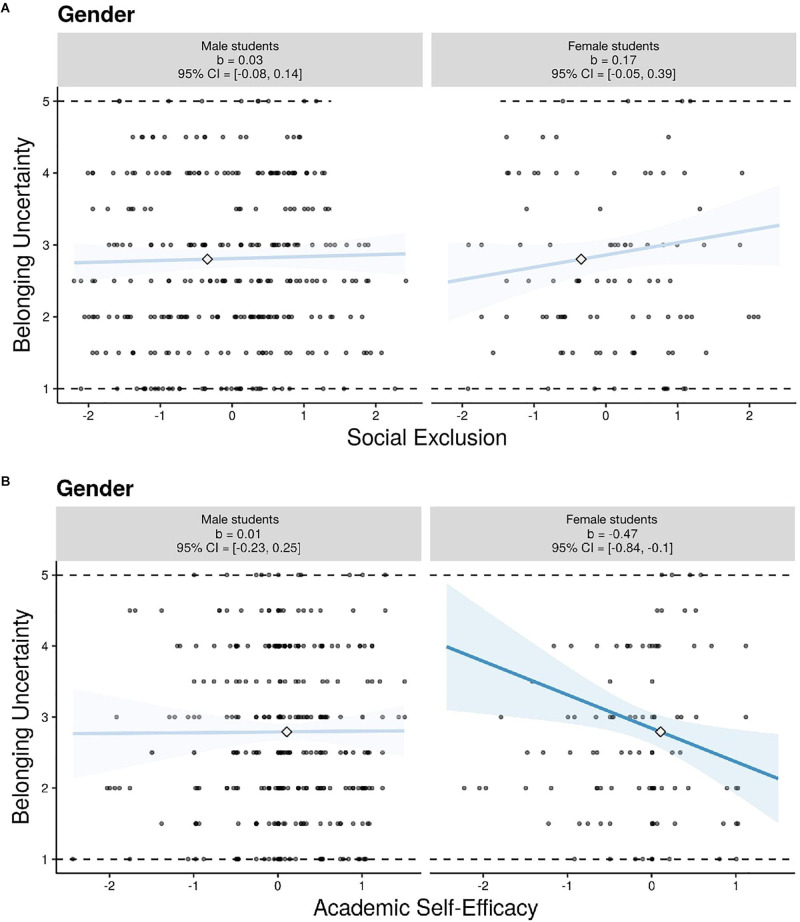
**(A)** Simple slopes graphs depicting the relationship between social exclusion and belonging uncertainty. Academic self-efficacy, relative potential, the initial level of belonging uncertainty, academic performance, gender, academic self-efficacy X gender, and relative potential X gender were entered as covariates. **(B)** Simple slopes graphs depicting the relationship between academic self-efficacy and belonging uncertainty. Social exclusion, relative potential, the initial level of belonging uncertainty, academic performance, gender, social exclusion X gender, and relative potential X gender were entered as covariates. In each panel, simple slopes are displayed for both levels of the moderator. Each graphic shows the computed 95% confidence region (shaded area), the observed data (gray circles), the minimum and maximum values of the outcome (dashed horizontal lines), and the crossover point (diamond). Regression coefficients (*b*) differ from the ones depicted in Table 3 due to full information maximum likelihood estimation (FIML) within Mplus. CI = confidence interval. Figures were produced using the interActive data visualization tool (McCabe et al., 2018).

In line with our first hypothesis, the interaction between students’ perceived affective and academic exclusion by fellow students and gender was a significant predictor of belonging uncertainty (β = 0.087, *p* = 0.038). Although there were no significant mean differences in the perceived affective and academic exclusion between male and female computer science students as depicted in Table 1, it was found to be a significant predictor of female students’ belonging uncertainty. Thus, female students with higher values of perceived social exclusion relative to their group mean experienced greater uncertainty about their belonging than their male peers (see Figure 1A). Consistent with our second hypothesis, the interaction between students’ domain-specific academic self-efficacy and gender was found to be predictive of the uncertainty about belonging in the domain of computer science (β = −0.133, *p* = 0.019). As expected, academic self-efficacy was a more relevant predictor of belonging uncertainty for female than for male students, i.e., female students with lower self-efficacy beliefs in relation to the average degree of academic self-efficacy beliefs in their respective tutorial group were more uncertain about their belonging in computer science than male students (see Figure 1B). Contrary to our expectations, the link between the perceived performance potential in comparison to fellow students and feelings of belonging uncertainty did not differ as a function of gender (β = 0.017, *p* = 0.742). Thus, the slopes of the regression lines did not differ significantly between male and female students. Rather, a significant main effect of the relative potential (β = −0.147, *p* = 0.005) indicated that the assessment of one’s capabilities through social comparisons with others in the same academic domain is a relevant predictor of both male and female students’ uncertainty about belonging. The overall model explained a total of 51.1% of the variance in the outcome variable, with Cohen’s *f*^2^ statistic yielding an effect size estimate of 1.04, which corresponds to a large effect (Cohen, 1988).

## Discussion

Female students in computer science, like in many other STEM domains, still constitute a numerical minority. Thus, an important goal for research in this field is to study the factors that prevent students from meeting their academic potential. A well-established predictor of minority students’ academic underachievement is the worry to not “fit into” the respective academic environment: belonging uncertainty. While much research has focused on the outcomes of belonging uncertainty, we sought to deepen our understanding of the sources of belonging uncertainty. We examined the role of (a) the perceived affective and academic exclusion by fellow students, (b) domain-specific academic self-efficacy beliefs, and (c) the perceived individual performance potential in comparison to that of fellow students as possible predictors of female students’ belonging uncertainty. In doing so, this study adds to the literature by extending our conceptual understanding of the uncertainty about belonging.

Consistent with our expectations, we found that perceived affective and academic exclusion by fellow students increased female but not male students’ belonging uncertainty in computer science. This finding conforms to the assumption that members of underrepresented and negatively stereotyped groups, as in the case of female students in STEM, are particularly sensitive to the quality of their social relationships in competitive academic environments (Walton and Cohen, 2007). Although male and female students did not differ in the extent to which they felt excluded from non-academic social activities and subject-related exchange, the subjective experience of being socially excluded was a relevant explanation for female students’ doubts whether they would belong. Thus, our results indirectly support

previous research on the cues hypothesis, which holds that subtle situational cues, such as the numerical representation of a social group, can signal a lack of social connectedness and trigger experiences of social identity threat among stereotyped groups (Murphy et al., 2007).

Further and in line with our predictions, we found domain-specific academic self-efficacy beliefs to be a significant predictor, again, of female but not male students’ uncertainty about belonging. Previous experimental studies have indicated the negative influence of entity beliefs and negative ability-related stereotypes on self-efficacy beliefs (Hoyt and Blascovich, 2007; Lerdpornkulrat et al., 2012; Wang et al., 2017), and our results seem to confirm such a link. Female students with a threatened social identity in the context of a male-dominated STEM subject appear to be more susceptible to low academic self-efficacy beliefs to the effect that they constitute a relevant source of their sense of belonging uncertainty.

Unexpected, however, was that the extent to which students felt they had lower academic potential than their fellow students was not more important for female students, but rather a relevant predictor of both male and female students’ uncertainty about belonging in computer science. Both male and female students’ upward social comparisons regarding their academic potential in computer science appear to affect their belonging uncertainty in a similar vein, regardless of the relative number of in- or out-group members. The presence of negative stereotypes about females’ abilities in the field may explain differences in male and female assessment of their relative performance potential (cf., Ertl et al., 2017). Yet, our pattern of results suggests that being a member of a potentially stereotyped minority does not imply that females weight their perceived potential more strongly than males when judging their belonging to the specific academic domain.

Belonging uncertainty has frequently been defined as concern about the quality of one’s social ties or about whether one would be fully included in positive social relationships (e.g., Walton and Cohen, 2007, 2011). Our findings that domain-specific academic self-efficacy beliefs as well as the perceived performance potential in comparison to peers are relevant sources of students’ uncertainty about belonging expand this conceptual view. This is in line with research by Walton and Cohen (2007) who found that female students’ sense of belonging was negatively affected by a manipulation that made them believe they had only a few of the skills required in computer science. It appears that belonging uncertainty is rooted in both students’ doubts about their social connectedness within an academic domain and concerns about whether they have the abilities to succeed in that domain.

Experimental research has previously suggested a difference between academic and social belonging (Skourletos et al., 2013). But rather than sources of one concept, academic and social belonging were considered two different types of belonging, each with a discriminative power in the prediction of achievement-related variables. Similarly, Lewis and Hodges (2015) developed a domain-specific measure of ability uncertainty and found negative correlations with social belonging and academic self-efficacy. In addition, the authors found ability uncertainty to predict academic outcomes, such as students’ intent to persist in their psychology or linguistics major. Again, ability fit and social fit within a particular academic domain were conceptualized as two separate types of belonging individuals might question. Interestingly, Lewis and Hodges (2015) examined a sample of psychology and linguistics students who were predominantly female and Caucasian and thus, did not have to contend with negative stereotypes about their intellectual ability within their domains. With the particular group of computer science students, in which females are a stereotyped minority, our results suggest that belonging uncertainty is in fact a result of their lower confidence in their abilities to overcome academic challenges, their feeling of being less competent than their – primarily male – peers, and their perception of being excluded from social and academic exchange with fellow students.

In summary, the results of the present research suggest that conceptualizing belonging uncertainty as the concerns about one’s social connectedness in an academic domain, without incorporating stereotyped students’ concerns about their academic abilities, and vice versa, might be an incomplete understanding of the uncertainty about belonging in an educational environment.

Although further research is needed to substantiate our findings, it is possible to consider practical implications for educational institutions in terms of how to organize the integration of minority students into their study programs and foster belonging. First, our results show that belonging uncertainty, a strong predictor of students’ achievement, persistence, and career aspirations (Woodcock et al., 2012; Cundiff et al., 2013; Walton et al., 2015; Höhne and Zander, in press), is catalyzed by perceived affective and academic social exclusion, particularly among female students. Therefore, study programs may wish to create more opportunities for male and female students to exchange with their peers – both formally, e.g., in specific group learning arrangements in seminars and tutorials, as well as informally, after classes. These activities may be especially relevant at the beginning of students’ university careers in order to prevent increasing disparities. Second, our findings suggest that academic self-efficacy beliefs are particularly important for female students’ sense of belonging. Given that successful models, credible social persuasion, experiences of mastery, and positive affective states are important sources of self-efficacy (Bandura, 1997; Bong and Skaalvik, 2003), pedagogical support and mentoring from lecturers and graduate students providing these sources may be a promising route to improve female students’ feelings of belonging in male-dominated STEM domains.

### Limitations and Future Directions

Despite the contribution of the present research to the understanding of female students’ uncertainty about belonging in the male-dominated STEM subject of computer science, our study has some limitations that need to be addressed in future research.

To begin with, our study is not experimental in nature, and thus, conclusions about the causal relationship between students’ perceived affective and academic exclusion by fellow students, their reported academic self-efficacy in computer science, their perceived performance potential in comparison to peers, and their uncertainty about belonging in computer science should be made with caution. This being said, the fact that our predictor variables were measured prior to belonging uncertainty, that students’ perceptions about their social exclusion and academic competencies at the beginning of the semester predicted belonging uncertainty in the midst of the semester, and that we controlled for the initial sense of belonging uncertainty suggest the directionality of the effect. The results of an experimental study by Walton and Cohen (2007) point in the same direction. Here, students were made to believe that they had only limited computer science skills, which lowered female students’ sense of belonging. This reasoning is further supported by a study in which Walton et al. (2015) applied an intervention to mitigate doubts about social belonging in engineering. A core element of the intervention was the implication of normality when experiencing doubts. Through written experiences of former first-year students, participants were told that almost all students had worries about fitting in and being accepted during their first year in college, but that these concerns would dissipate with time. The authors found that the intervention helped female students to better integrate in their engineering study program and establish friendships with male students (Walton et al., 2015). This suggests that social doubts precede belonging uncertainty, rather than resulting from it. Future experimental research or longitudinal designs with cross-lagged analyses could help to further clarify the interrelation between these constructs.

Second, the present study is limited in that it only examined a sample of undergraduate computer science students. It would be an important next step to examine whether this pattern of findings can be applied to female students in other STEM domains and to other social groups that have to contend with negative ability-related stereotypes and who constitute a numerical minority in their respective academic domain. Moreover, longitudinal studies over a longer period of time are needed to investigate the temporal stability of our findings. With regard to the subject of computer science, we decided to assess students at the beginning and in the midst of their first semester because of the high dropout rates in this subject at German universities (Heublein and Schmelzer, 2018) and because previous research could show that doubting one’s belongingness in an academic context itself is a predictor of students’ persistence and dropout intentions, respectively (Woodcock et al., 2012; Cundiff et al., 2013; Höhne and Zander, in press). Therefore, and because we wanted to investigate the sources of belonging uncertainty, we expected a certain proportion of the students with a high uncertainty about belonging to already have dropped out by the end of the semester, preventing us from obtaining insight into the psychological experiences of this student group. However, studies over a longer period of time would not only advance our theoretical understanding of the concept of belonging uncertainty, but also inform interventions about when they can exert maximal effects on members of affected social groups in different academic domains. Additionally, if we assume that the sources of belonging uncertainty hold for other academic settings and stereotyped social groups, then a positive sense of belonging could stem from the same sources, but with different signs. Considering empirical support for the phenomenon of stereotype lift, i.e., a performance boost caused by downward comparisons with members of a negatively stereotyped out-group (for a meta-analysis see Walton and Cohen, 2003), it is plausible that similar mechanisms underlie the development of positive belongingness.

Third, although our finding that belongingness to an academic domain is depending on the perception of one’s social and ability fit is in line with the results of a study by Lewis and Hodges (2015), who found a significant correlation between ability uncertainty and social belonging, there may be other components that add to the full picture of belonging uncertainty. For example, research in the field of computer science could demonstrate that girls show an increased interest and sense of belonging when introducing them to physical environments that were not considered stereotypical of computer science (Master et al., 2016). Classrooms and other physical environments that signal a stereotypical image of computer science and the people that represent that domain might therefore result in an adverse balance of self-to-prototype matching in female students and, in turn, serve as source of feelings of belonging uncertainty in that domain. Future research is needed to systematically study how adaptive processes of group formation can be initiated and stimulated through learning arrangements and how institutional norms can further contribute to create a learning environment supportive for all students, including those constituting a minority.

A fourth limitation involves methodological issues. It should be noted that the present research is restricted to self-reports and thus reflects students’ perceptions of their degree of social inclusion and academic competencies, rather than information from external sources, such as students’ actual test grades as a measure of academic performance in college and the reports of other peers, teachers, or even observational data regarding their social inclusion. Another methodological limitation concerns our measures. Given that we conducted research in a real-life educational setting with considerable time constraints, some of the scales applied only consisted of a limited number of items. However, it would be desirable to use multi-item measures in future research in order to provide stronger validity to the present results and to develop new measures of students’ sense of belonging that take into account both the social and the ability-related component of the construct. Further, to tap into the social component of the ability measure, we used a one-item measure applied by Walton and Cohen (2007), asking students to evaluate their individual performance potential in comparison to that of fellow students on a percentile scale. Given the minority situation of female students, it would have been interesting to add a measure tapping into their perceived performance compared to other female or male students, respectively, and to assess which student group the relevant group of reference is. Another limitation possibly related to the application of few-item scales in our study concerns the weak to moderate correlations between some of our independent variables, especially with regard to the correlation between our measures of academic self-efficacy and the perceived individual performance potential compared to that of fellow students. Although both concepts are theoretically clearly distinct from each other, they overlap in that both describe perceptions of the self in academic contexts. Here, again, it would be desirable to apply multi-item scales to enhance the predictive validity of the measures and further, to conduct confirmatory factor analyses to quantify the extent of conceptual overlap in future studies. Lastly, disparities in gender representation also became apparent in our study: of our total sample, only 23% were female computer science students, resulting in a relative small share of our focal group of participants in the sample. Although we have a representative sample in terms of gender in our study (Federal Statistical Office of Germany, 2018), the naturally larger standard error in the smaller subgroup makes our testing more conservative, with the result that we might even underestimate gender mean differences (see Gelman and Hill, 2007). Moreover, in moderated multiple regression analyses with unequal subgroup sample sizes, the statistical power of the inferential test cannot exceed the power of a test involving two subgroups, each of the size of 2(n_1_) with n_1_ being the size of the smaller subgroup, regardless of the size of the second subgroup (Arguinis, 2004). The large – and typical – dropout numbers in computer science further contribute to this problem. Future research replicating our findings with larger sample sizes would therefore be desirable and important.

## Conclusion

As female students face negative stereotypes about their ability in quantitative fields and continue to remain underrepresented in computer science, understanding the factors that explain these phenomena is key for creating stimulating and encouraging educational environments for females studying a male-dominated subject such as computer science. When considering the causes and cures of this existing gender gap, students’ uncertainty about belonging is a promising variable to study.

The present study identified male and female sources of belonging uncertainty in the computer sciences and thereby extends our understanding of this theoretical concept. Our results suggest that belonging uncertainty is comprised of both students’ concerns about their social connectedness in an academic domain and concerns about their academic abilities. Therefore, conceptualizing belonging uncertainty as regarding only concerns about the quality of one’s social relationships in an academic domain leads to an incomplete picture of this phenomenon.

By identifying the sources of the uncertainty about belonging in computer science, our results may serve to inform the institutional organization of minority students’ integration into their studies as well as interventions aimed at fostering students’ sense of belonging and increasing the share of female students in computer science and other STEM domains.

## Ethics Statement

According to our university guidelines and to national regulations in Germany, no ethics review was required, because the current research can be classified as research using anonymous or no-risk tests, surveys, interviews, or observations. Written informed consent was obtained from all participants prior to every data assessment.

## Author Contributions

LZ provided the initial idea, conceived, and designed the study. EH conducted the data collection, organized the data base, carried out the statistical analyses, and wrote the first draft of the manuscript. Both authors contributed to the manuscript revision, read, and approved the submitted version of the manuscript.

## Conflict of Interest Statement

The authors declare that the research was conducted in the absence of any commercial or financial relationships that could be construed as a potential conflict of interest.

## Footnotes

1 Although double-barreled items like this one should generally be avoided because respondents could hold different views about different topics within one item (Simms and Watson, 2007), the authors specifically aimed at measuring students’ doubts or uncertainty about their belonging within computer science (cf., Walton and Cohen, 2007).

2 Noteworthy, Cronbach’s alpha is an estimate of reliability under rather restrictive assumptions, wherefore the coefficient almost always underestimates true reliability (Bollen, 1989; Sijtsma, 2009; Eisinga et al., 2013).

3 Standardized mean differences were calculated using Cohen’s *d* statistic, with small, medium, and large effect sizes (ES) being equivalent to *d*-values of 0.20, 0.50, and 0.80, respectively (Cohen, 1988). ES were calculated using the following formula: $\Delta =\frac{\beta_{1}}{\sigma_{e}}$ (Tymms et al., 1997).

4 We expected the relevant reference group for participants to be students in their tutorial group, rather than all students in their study program, because we conducted our study in the tutorial classes of the first-year students. Hence, we expected students to be not yet familiar with many of their fellow students. In contrast to the teacher-centered teaching in lectures, students have to actively take part in the smaller and compulsory tutorial classes, e.g., by handing in weekly exercises that they worked on in groups, thereby getting to know each other faster. We therefore expected students to assess e.g., their performance potential and their affective as well as academic exclusion in relation to the students in their tutorial group.
