# Corrigendum: Sources of male and female students' belonging uncertainty in the computer sciences

**DOI:** 10.3389/fpsyg.2022.1096269

**Published:** 2023-02-01

**Authors:** Elisabeth Höhne, Lysann Zander

**Affiliations:** Division of Empirical Educational Research, Institute of Education, Leibniz Universität Hannover, Hanover, Germany

**Keywords:** belonging uncertainty, ability-related stereotypes, social identity, minority students, higher education, STEM, computer science, gender

In the published article, there was an error in the **Conclusion**, Paragraph 2. In the final sentence “relationship” should have read “relationships.” The corrected paragraph appears below.

“The present study identified male and female sources of belonging uncertainty in the computer sciences and thereby extends our understanding of this theoretical concept. Our results suggest that belonging uncertainty is comprised of both students' concerns about their social connectedness in an academic domain and concerns about their academic abilities. Therefore, conceptualizing belonging uncertainty as regarding only concerns about the quality of one's social relationships in an academic domain leads to an incomplete picture of this phenomenon.”

In addition, there were also errors in some of the references in the published article.

The reference for Berkman and Syme (1979) was written as “Berkman, L. F., and Syme, L. (1979). Social networks, host resistance, and mortality: a nine-year follow-up study of Alameda county residents. *Am. J. Epidemiol*. 109, 186–204. doi: 10.1017/CBO9780511759048” It should be “Berkman, L. F., and Syme, S. L. (1979). Social networks, host resistance, and mortality: a nine-year follow-up study of Alameda county residents. *Am. J. Epidemiol*. 109, 186–204. doi: 10.1093/oxfordjournals.aje.a112674”.

The reference for Cohen (1988) was written as: “Cohen, J. E. (1988). *Statistical Power Analysis for the Behavioral Sciences*, 2nd Edn. Hillsdale, NJ: Lawrence Erlbaum Associated, Inc.” It should be “Cohen, J. (1988). *Statistical Power Analysis for the Behavioral Sciences*, 2nd Edn. Hillsdale, NJ: Lawrence Erlbaum Associates, Inc.”

The reference for Eisinga et al. (2013) was written as “Eisinga, R., te Grotenhuis, M., and Pelzer, B. (2013). The reliability of a two-item scale: Pearson, Cronbach, or Spearman-Brown? *Int. J. Public Health* 58, 637–643. doi: 10.1007/s00038-012-0416-3” It should be “Eisinga, R., te Grotenhuis, M., and Pelzer, B. (2013). The reliability of a two-item scale: Pearson, Cronbach, or Spearman-Brown? *Int. J. Public Health* 58, 637–642. doi: 10.1007/s00038-012-0416-3”.

The reference for Marsh and O'Mara (2008) was incorrectly written as “Marsh, H. W., and O'Mara, A. (2006). Reciprocal effects between academic self-concept, self-esteem, achievement, and attainment over seven adolescent years: unidimensional and multidimensional perceptions of self-concept. *Pers. Soc. Psychol. Bull*. 34, 542–552. doi: 10.1177/0146167207312313” It should be “Marsh, H. W., and O'Mara, A. (2008). Reciprocal effects between academic self-concept, self-esteem, achievement, and attainment over seven adolescent years: unidimensional and multidimensional perspectives of self-concept. *Pers. Soc. Psychol. Bull*. 34, 542–552. doi: 10.1177/0146167207312313”.

The reference for Marsh et al. (2019) was incorrectly written as “Marsh, H. W., Pekrun, R., Parker, P. D., Murayama, K., Guo, J., Dicke, T., et al. (2018). The murky distinction between self-concept and self-efficacy: beware of lurking jingle-jangle fallacies. *J. Educ. Psychol*. 111, 331–353. doi: 10.1037/edu0000281” It should be “Marsh, H. W., Pekrun, R., Parker, P. D., Murayama, K., Guo, J., Dicke, T., et al. (2019). The murky distinction between self-concept and self-efficacy: beware of lurking jingle-jangle fallacies. *J. Educ. Psychol*. 111, 331–353. doi: 10.1037/edu0000281”.

The reference for Master et al. (2016) was incorrectly written as “Master, A., Cheryan, A., and Meltzoff, A. N. (2016). Computing whether she belongs: stereotypes undermine girls' interest and sense of belonging in computer science. *J. Educ. Psychol*. 108, 424–437. doi: 10.1037/edu0000061” It should be “Master, A., Cheryan, S., and Meltzoff, A. N. (2016). Computing whether she belongs: stereotypes undermine girls' interest and sense of belonging in computer science. *J. Educ. Psychol*. 108, 424–437. doi: 10.1037/edu0000061”.

The reference for Murphy and Taylor (2012) was incorrectly written as “Murphy, M. C., and Taylor, V. J. (2012). “The role of situational cues in signaling and maintaining stereotype threat,” in *Stereotype Threat: Theory, Process, Application*, eds M. Inzlicht and T. Schmader (New York, NY: Oxford University Press), 17–33.” It should be “Murphy, M. C., and Taylor, V. J. (2012). “The role of situational cues in signaling and maintaining stereotype threat,” in *Stereotype Threat: Theory, Process, and Application*, eds M. Inzlicht and T. Schmader (New York, NY: Oxford University Press), 17–33.”

The reference for Robbins et al. (2004) was incorrectly written as “Robbins, S. B., Lauver, K., Le, H., Davis, D., and Langley, R. (2004). Do psychosocial and study skill factors predict college outcomes? A meta-analysis. *Psychol. Bull*. 130, 261–288. doi: 10.1037/0033-2909.130.2.261” It should be “Robbins, S. B., Lauver, K., Le, H., Davis, D., Langley, R., and Carlstrom, A. (2004). Do psychosocial and study skill factors predict college outcomes? A meta-analysis. *Psychol. Bull*. 130, 261–288. doi: 10.1037/0033-2909.130.2.261”.

The reference for Sijtsma (2009) was incorrectly written as “Sijtsma, K. (2009). On the use, misuse, and the very limited usefulness of Cronbach's alpha. *Psychometrika* 74, 107–120. doi: 10.1007/s11336-008-9101-0” It should be “Sijtsma, K. (2009). On the use, the misuse, and the very limited usefulness of Cronbach's alpha. *Psychometrika* 74, 107–120. doi: 10.1007/s11336-008-9101-0”.

The reference for Simms and Watson (2007) was incorrectly written as “Simms, L. J., and Watson, D. (2007). “The construct validation approach to personality scale construction,” in *Handbook of Research Methods in Personality Psychology*, eds R. W. Robins, R. C. Fraley, and R. F. Kruger (New York, NY: Guilford Press), 240–258.” It should be “Simms, L. J., and Watson, D. (2007). “The construct validation approach to personality scale construction,” in *Handbook of Research Methods in Personality Psychology*, eds R. W. Robins, R. C. Fraley, and R. F. Krueger (New York, NY: Guilford Press), 240–258.”

The reference for Tajfel and Turner (1986) was incorrectly written as “Tajfel, H., and Turner, J. C. (1986). “The social identity theory of intergroup behavior,” in *Psychology of Intergroup Relations*, eds S. Worchel and L. W. Austin (Chicago: Nelson Hall), 7–24.” It should be “Tajfel, H., and Turner, J. C. (1986). “The social identity theory of intergroup behavior,” in *Psychology of Intergroup Relations*, eds S. Worchel and W. G. Austin (Chicago: Nelson Hall), 7–24.”

The reference for Tymms et al. (1997) was incorrectly written as “Tymms, P., Merrell, C., and Henderson, B. (1997). The first at school: a quantitative investigation of the attainment and progress of pupils. *Educ. Res. Eval*. 3, 101–118. doi: 10.1080/1380361970030201” It should be “Tymms, P., Merrell, C., and Henderson, B. (1997). The first year at school: a quantitative investigation of the attainment and progress of pupils. *Educ. Res. Eval*. 3, 101–118. doi: 10.1080/1380361970030201”.

The reference for Walton and Carr (2012) was incorrectly written as “Walton, G. M., and Carr, P. B. (2012). “Social belonging and the motivation and intellectual achievement of negatively stereotyped students,” in *Stereotype Threat: Theory, Process, Application*, eds M. Inzlicht and T. Schmader (New York, NY: Oxford University Press), 89–106.” It should be “Walton, G. M., and Carr, P. B. (2012). “Social belonging and the motivation and intellectual achievement of negatively stereotyped students,” in *Stereotype Threat: Theory, Process, and Application*, eds M. Inzlicht and T. Schmader (New York, NY: Oxford University Press), 89–106.”

The reference for Woodcock et al. (2012) was incorrectly written as “Woodcock, A., Hernandez, P. R., Estrada, M., and Schultz, P. W. (2013). The consequences of chronic stereotype threat: domain disidentification and abandonment. *J. Pers. Soc. Psychol*. 103, 635–646. doi: 10.1037/a0029120” It should be “Woodcock, A., Hernandez, P. R., Estrada, M., and Schultz, P. W. (2012). The consequences of chronic stereotype threat: domain disidentification and abandonment. *J. Pers. Soc. Psychol*. 103, 635–646. doi: 10.1037/a0029120”.

Finally, an amendment has been made to footnote 4 in the original article. The first sentence previously stated: “We expected the relevant reference group for participants to be students in their tutorial group, rather all students in their study program, because we conducted our study in the tutorial classes of the first-year students.” This should be: “We expected the relevant reference group for participants to be students in their tutorial group, rather than all students in their study program, because we conducted our study in the tutorial classes of the first-year students.” The corrected footnote is found below:

We expected the relevant reference group for participants to be students in their tutorial group, rather than all students in their study program, because we conducted our study in the tutorial classes of the first-year students. Hence, we expected students to be not yet familiar with many of their fellow students. In contrast to the teacher-centered teaching in lectures, students have to actively take part in the smaller and compulsory tutorial classes, e.g., by handing in weekly exercises that they worked on in groups, thereby getting to know each other faster. We therefore expected students to assess e.g., their performance potential and their affective as well as academic exclusion in relation to the students in their tutorial group.

The authors apologize for these errors and state that they do not change the scientific conclusions of the article in any way. The original article has been updated.

