# Supplementary material for: Sources of Male and Female Students’ Belonging Uncertainty in the Computer Sciences
Source: Front Psychol. 2019 Aug 13;10:1740. doi: 10.3389/fpsyg.2019.01740 (PMC6700275; doi:10.3389/fpsyg.2019.01740)
Supplement: Supplementary file 1 [file Data_Sheet_1.PDF]

Supplementary Material

1 Measures

1.1 Belonging uncertainty (adapted from Walton & Cohen, 2011).

	strongly disagree	disagree	neither	agree	strongly agree
Sometimes I feel that I belong to this study program, and sometimes I feel that I don't belong to this study program.	☐	☐	☐	☐	☐
When things don't go well, I often think that maybe I don't belong to this study program.	☐	☐	☐	☐	☐

1.2 Affective and academic exclusion by fellow students (self-developed).

Sometimes I have the feeling …	strongly disagree	disagree	neither	agree	strongly agree
… that other students meet privately and I am not included.	☐	☐	☐	☐	☐
… that other students engage in subject-related exchange and I am not included.	☐	☐	☐	☐	☐
I have already noticed …	strongly disagree	disagree	neither	agree	strongly agree
… that other students get along with each other better than it is the case for me.	☐	☐	☐	☐	☐
… that other students engage in subject-specific exchange and I am not included.	☐	☐	☐	☐	☐

1.3 Domain-specific academic self-efficacy (adapted from Jerusalem & Schwarzer, 1986).

	strongly disagree	disagree	neither	agree	strongly agree
I am confident that I have the competencies to perform well in this subject.	☐	☐	☐	☐	☐
I can cope with difficult situations and challenges in my studies when I try hard.	☐	☐	☐	☐	☐

1.4 Performance potential compared to fellow students (adapted from Walton & Cohen, 2007).

Now, think of the students in your subject. How much potential – compared to your fellow students – do you have to succeed in your studies? Please tick only one of the boxes. I have more potential than … of the students in this subject.								
10%	20%	30%	40%	50%	60%	70%	80%	90%
☐	☐	☐	☐	☐	☐	☐	☐	☐

2 Statistical analyses

On request of one of the reviewers, we provide the full regression equation of our main analysis:

$$\hat{y} = b_0 + b_1x_1 + b_2x_2 + b_3x_3 + b_4x_4 + b_5x_5 + b_6x_6 + b_7x_1x_6 + b_8x_2x_6 + b_9x_3x_6$$

$$\hat{y} = b_0 + b_1\, Social\ exclusion + b_2\, Academic\ self-efficacy + b_3\, Relative\ potential + b_4\, Belonging\ uncertainty + b_5\, Academic\ performance + b_6\, Gender + b_7\, Social\ exclusion \times gender + b_8\, Academic\ self-efficacy \times gender + b_9\, Relative\ potential \times gender$$

$$\hat{y} = 2.793 - 0.014\, Social\ exclusion - 0.063\, Academic\ self-efficacy - 0.147\, Relative\ potential + 0.561\, Belonging\ uncertainty - 0.074\, Academic\ performance + 0.011\, Gender + 0.087\, Social\ exclusion \times gender - 0.133\, Academic\ self-efficacy \times gender + 0.017\, Relative\ potential \times gender$$

3 Supplementary tables

Supplementary Table 1

Hierarchical linear regression for variables at T1 predicting belonging uncertainty at T2

	B	***SE***	***β***	***R^2***	***F for change in R^2***
**Model 1**					
Social exclusion	.211	.051	.201***	.040	18.625***
**Model 2**					
Social exclusion	.128	.046	.120**		
Academic self-efficacy	-.739	.071	-.464***	.246	121.851***
**Model 3**					
Social exclusion	.136	.044	.128**		
Academic self-efficacy	-.476	.099	-.301***		
Relative potential	-.163	.034	-.284***	.295	30.929***

**Model 4**					
Social exclusion	.029	.045	.028		
Academic self-efficacy	-.212	.087	-.135*		
Relative potential	-.075	.028	-.131**		
Belonging uncertainty	.536	.040	.553***	.497	178.306***
**Model 5**					
Social exclusion	.028	.047	.026		
Academic self-efficacy	-.215	.087	-.136*		
Relative potential	-.077	.027	-.135**		
Belonging uncertainty	.536	.039	.554***		
Academic performance	-.043	.121	-.063	.499	1.768
**Model 6**					
Social exclusion	.026	.047	.025		
Academic self-efficacy	-.216	.084	-.137**		
Relative potential	-.074	.028	-.130**		
Belonging uncertainty	.534	.041	.552***		
Academic performance	-.042	.123	-.062		
Gender	.068	.139	.026	.501	1.772
**Model 7**					
Social exclusion	-.029	.046	-.027		
Academic self-efficacy	-.215	.082	-.137**		
Relative potential	-.076	.027	-.133**		
Belonging uncertainty	.542	.039	.563***		
Academic performance	-.042	.115	-.061		
Gender	.036	.132	.014		
Social exclusion X gender	.239	.101	.101*	.504	2.667
**Model 8**					
Social exclusion	-.015	.045	-.014		
Academic self-efficacy	-.109	.080	-.069		
Relative potential	-.078	.026	-.137**		
Belonging uncertainty	.540	.037	.561***		
Academic performance	-.042	.115	-.062		
Gender	.025	.127	.009		
Social exclusion X gender	.207	.094	.088*		
Academic self-efficacy X gender	-.390	.135	-.122**	.510	5.388*
**Model 9**					
Social exclusion	-.015	.045	-.014		
Academic self-efficacy	-.099	.082	-.063		
Relative potential	-.084	.030	-.147		
Belonging uncertainty	.540	.036	.561		
Academic performance	-.050	.123	-.074		
Gender	.029	.129	.011		
Social exclusion X gender	.204	.096	.087		
Academic self-efficacy X gender	-.424	.175	-.133		
Relative potential X gender	.019	.058	.017	.511	0.898

Notes. N = 449. All values were estimated using Mplus and full information maximum likelihood estimation (FIML). Standard errors were corrected for hierarchical data structure. *SE* = standard error of *B*. Gender: 0 = male, 1 = female. *$p \leq .05$. **$p \leq .01$. ***$p \leq .001$.
